# A Study on the Reliability of Sasang Constitutional Body Trunk Measurement

**DOI:** 10.1155/2012/604842

**Published:** 2011-07-21

**Authors:** Eunsu Jang, Jong Yeol Kim, Haejung Lee, Honggie Kim, Younghwa Baek, Siwoo Lee

**Affiliations:** ^1^Department of Sasang Constitutional Medicine, Korea Institute of Oriental Medicine, Daejeon 305811, Republic of Korea; ^2^Department of Information and Statistics, Chungnam National University, Daejeon, Republic of Korea

## Abstract

*Objective*. Body trunk measurement for human plays an important diagnostic role not only in conventional medicine but also in Sasang constitutional medicine (SCM). The Sasang constitutional body trunk measurement (SCBTM) consists of the 5-widths and the 8-circumferences which are standard locations currently employed in the SCM society. This study suggests to what extent a comprehensive training can improve the reliability of the SCBTM. *Methods*. We recruited 10 male subjects and 5 male observers with no experience of anthropometric measurement. We conducted measurements twice before and after a comprehensive training. Relative technical error of measurement (%TEMs) was produced to assess intra and inter observer reliabilities. *Results*. Post-training intra-observer %TEMs of the SCBTM were 0.27% to 1.85% reduced from 0.27% to 6.26% in pre-training, respectively. Post-training inter-observer %TEMs of those were 0.56% to 1.66% reduced from 1.00% to 9.60% in pre-training, respectively. Post-training % total TEMs which represent the whole reliability were 0.68% to 2.18% reduced from maximum value of 10.18%. *Conclusion*. A comprehensive training makes the SCBTM more reliable, hence giving a sufficiently confident diagnostic tool. It is strongly recommended to give a comprehensive training in advance to take the SCBTM.

## 1. Introduction

In Western society, scientific interest has been focused on the body trunk measurement in association with disease. Body mass index (BMI) has drawn popularity as a predictor of the metabolic disease [1] and people with larger waist circumference (WC) are prone to this disease even when the BMI is not high [2]. Recently, waist-to-hip ratio (WHR) was found to have association with the colon cancer as well as to be a risk factor for heart disease [3, 4]. Second and fourth digital length ratio (2D : 4D) is known to have association with the attention-deficit hyperactivity disorder (ADHD) or cardiovascular diseases such as myocardial infarction [5, 6]. Like these, ratios between specific parts of the body are an important index of disease prediction. 

In traditional Korean medicine, individual's constitutional type is regarded as being related to body shape, psychological status, physiological function, and susceptibility to diseases [7, 8]. Some research reported that physique characteristics and disease prevalence are different from one constitution to another [9]. Especially regarding body shape, digestive system is hypoactive in individuals with small chest, excretion and sexual function are weak if one's hip area is small, and lung hypofunction is related with big waist circumference [10, 11]. Clinically, the same disease may be treated differently according to patient's constitution and different diseases are sometimes treated with the same prescription. Above all, the Sasang constitutional medicine (SCM) specialists focus on determining patient's constitution correctly [12].

To determine individual's constitution, they examine temperament, characteristics of face, voice, body shape, physiological and pathological syndromes, and disease characteristics. Among these factors, the Sasang constitutional body trunk measurement (SCBTM) which consists of the 5-widths and the 8 circumferences plays a pivotal role in determining the constitution. The SCBTM is the standard location currently employed in the Korean SCM society [13, 14]. Thus, the correct measurement is very important in order to be a sufficiently confident diagnostic tool.

Reliability of anthropometric measurements such as WHR and WC was verified in several researches with multicenter data [15, 16] and other studies investigated intraobserver errors with the anthropometric methods of specific body areas [17, 18]. Measurement reliability to diagnose a specific disease was examined in the various researches, and several methods to improve measurement were suggested. Even though Korea's SCM specialists have used the SCBTM to determine individual's constitution, no studies have investigated whether they have sufficient reliability as diagnostic tool and how to improve the reliability of the SCBTM. 

This study suggests to what extent a comprehensive training can improve the reliability of the SCBTM.

## 2. Material and Methods

### 2.1. Study Design

We conducted this experiment from the first November to the third November, 2010 in Korea Institute of Oriental Medicine (KIOM) in Daejeon, Republic of Korea.

We recruited 10 male subjects and 5 male observers with no experience of anthropometric measurement. We also obtained informed consents from all participants.

This experiment was conducted for three different days. 

On the first day of experiment, as a pretraining session, the instructor at KIOM introduced the goal, schedule, and content of the experiment to subjects and observers. The instructor also showed photographs of the measuring points and documents explained the locations of the SCBTM to the observers. 

All subjects were respectively separated in the ten independent rooms randomly and observers including when the instructor got in the room to measure one by one. Five observers and the instructor measured ten subjects twice in order. Observers were advised not to share the measurements with other observers and submitted the assessment sheet to the instructor right after each subject's measuring was finished. 

On the second day, as a training session, a comprehensive training (Table 1) for the SCBTM, including the instructor's explanation, a couple of practices, and discussion, was conducted. 

This training has been used for the “Constitutional Clinical Information Collecting System” based on the standard body measurement research [19, 20]. Society of SCM is also using this manual for the measurements training in the traditional hospital too. 

The instructor explained and trained all the 5 observers about SCBTM and they were allowed to have two rounds of practical measuring. After practice, the instructor and the observers gathered together and discussed the locations of the SCBTM (duration of the training session: 4 hours). 

On the third day, as a posttraining session, all subjects were respectively separated in the independent rooms and 6 observers including instructor collected data from them with the same method used as in the first pretraining session.

### 2.2. General Characteristics

Participants consisted of subjects (*n* = 10) with the following characteristics: age, 24.4 years; weight, 74.16 kg; height, 178.42 cm, and observers (*n* = 5) with the following characteristics: age, 25.1 years; weight, 67.42 kg; height, 171.38 cm (Table 2).

The expert has been an instructor of KIOM and has been measuring the widths and circumferences of human body trunk for more than 5 years.

### 2.3. Measurements

The SCBTM consists of 5 widths and 8 circumferences. For the width measurement, interaxillary width (IW), chest width (CW), rib width (RW), waist width (WW), and pelvic width (PW) were measured. Locations of circumference measurement included forehead circumference (FC), neck circumference (NC), axillary circumference (AC), chest circumference (CC), rib circumference (RC), waist circumference (WC), pelvic circumference (PC), and hip circumference (HC).

Six large sliding calipers (50 cm/20 inch, Samhwa, Korea) and 6 tapelines (150 cm/60 inch, Hoechstmass, Germany) were used to measure the body width and circumference.

### 2.4. Statistical Analysis

Reliability was analyzed by the technical error of measurement (TEM), relative TEM, total TEM, and relative total TEM [21]. The TEM is the most commonly used measure of precision which is the square root of measurement error variance. Intraobserver TEM is estimated from differences between replicated measurements taken by one observer while interobserver TEM is estimated from measurements taken by 5 observers. 

Intraobserver TEM for one observer is calculated by

(1) $$\text{TEM}=\sqrt{\frac{\left(\sum D^{2}\right)}{2N}},$$

where *D* is the difference between measurements recorded by a given observer and *N* is the number of subjects measured. Interobserver TEM for five observers is calculated by

(2) $$\text{TEM}=\sqrt{\left(\frac{\left(\sum_{1}^{N}\left(\left(\sum_{1}^{K}M^{2}\right)-\left({\left(\sum_{1}^{K}M\right)}^{2}/K\right)\right)\right)}{N\left(K-1\right)}\right)},$$

where *N* is the number of subjects, *K* is the number of observers, and *M* is the measurement. 

The unit of TEM is the same as the unit of the measurement. Smaller TEM values represent more accurate measurement. The relative TEM (%TEM) is obtained by dividing the TEM for a given variable by the grand mean of that variable. 

To evaluate overall measurement variation, we calculated total TEM which includes both intra- and inter-observer TEM. A total TEM for five observers is calculated by

(3) $$\text{total}\;\text{TEM}=\sqrt{\left(\left(\frac{\left(\sum_{1}^{K}\text{TEM}{\left(\text{intra}\right)}^{2}\right)}{K}\right)+\text{TEM}{\left(\text{inter}\right)}^{2}\right)},$$

where *K* is the number of observer, TEM (intra) is the intraobserver TEM for the each observer and TEM(inter) is the interobserver TEM between observers. Relative total TEM (% total TEM) was calculated by

(4) $$\text{\%total}\;\text{TEM}=\left(\frac{\left(\text{total}\;\text{TEM}\right)}{\text{mean}}\right)\times 100.$$

In terms of reliability, smaller percentage represents more precise measurement. A %TEM score greater than 5% is usually considered imprecise in practical applications. 

The accuracy of the measurements of each observer was evaluated through the average bias (AB). AB was computed as a mean difference between measurement of observer and that of the instructor, which was designated as a gold standard due to his clinical experience and role as a trainer for the body shape measuring. Statistical analysis was performed using SPSS (Statistical Package for Social Sciences version 12.0; SPSS Inc., Chicago, Illinois, USA).

## 3. Results

### 3.1. Intraobserver Reliability

Five observers' pretraining and posttraining measurements are shown in Table 3. The range of intraobserver %TEMs in 5-width measurements ranged from 1.97%–4.98% pretraining and 0.79%–1.85% posttraining (IW), from 1.91%–6.26% pretraining and 1.05%–1.60% posttraining (CW), from 1.42%–3.48% pretraining and 0.77%–1.47% posttraining (RW), from 1.64%–2.78% pretraining and 0.77%–1.72% posttraining (WW), from 1.99%–5.71% pretraining and 0.58%–1.37% posttraining (PW).

The range of intraobserver %TEMs in 8-circumference measurements ranged from 0.27%–1.35% pretraining and 0.27%–0.54% posttraining (FC), from 1.04%–2.69% pretraining and 0.74%–0.98% posttraining (NC), from 1.09%–2.10% pretraining and 0.46%–1.03% posttraining (AC), from 1.16%–2.20% pretraining and 0.41%–0.67% posttraining (CC), from 1.28%–3.81% pretraining and 0.54%–1.35% posttraining (RC), from 0.91%–1.62% pretraining and 0.57%–0.83% posttraining (WC), from 0.87%–2.00% pretraining and 0.48%–1.10% posttraining (PC), from 1.27%–3.43% pretraining and 0.35%–0.99% posttraining (HC). Intraobserver %TEM of WHR using WC and HC ranged from 2.12%–3.10% pretraining and 0.70%–1.05% posttraining.

The %TEMs and AB values of the 5-width and 8-circumference measurements were decreased. This means that measurement differences between observers and instructor became smaller and the measurement reliability was increased.

### 3.2. Interobserver Reliability

The interobserver %TEMs in 5-width measurements were 4.53% pretraining and 0.82% posttraining (IW), 4.22% pretraining and 1.66% posttraining (CW), 2.40% pretraining and 1.12% posttraining (RW), 3.78% pretraining and 1.31% posttraining (WW), and 9.60% pretraining, and 1.62% posttraining (PW). 

The interobserver %TEMs in 8-circumference measurements were 1.00% pretraining and 0.56% posttraining (FC), 2.19% pretraining and 1.08% posttraining (NC), 1.80% pretraining and 1.05% posttraining (AC), 1.40% pretraining and 0.85% posttraining (CC), 1.75% pretraining and 0.98% posttraining (RC), 1.47% pretraining and 0.87% posttraining (WC), 2.08% pretraining and 1.33% posttraining (PC), and 2.43% pretraining and 0.88% posttraining (HC) (Table 4). Decreased %TEMs means that observer's performance is improved after the training session.

### 3.3. Intraobserver Repeatability and Interobserver Reliability

Intra- and inte-robserver reliabilities were integrated into % total TEM values, which was compared to assess total reliability of the measurement. 

The % total TEM values of the 5-width measurements were 5.65% pretraining and 1.53% posttraining (IW), 5.53% pretraining and 2.18% posttraining (CW), 3.60% pretraining and 1.62% posttraining (RW), 4.32% pretraining and 1.78% posttraining (WW), and 10.18% pretraining and 1.96% posttraining (PW).

**Figure 1:**
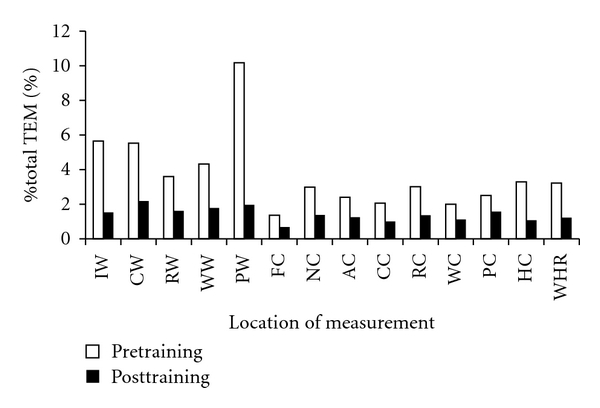
Pre- and post-training % total TEM of the measurement.

The % total TEM values of the 8-circumference measurements were 1.36% pretraining and 0.68% posttraining (FC), 2.99% pretraining and 1.38% posttraining (NC), 2.41% pretraining and 1.25% posttraining (AC), 2.06% pretraining and 1.01% posttraining (CC), 3.01% pretraining and 1.36% posttraining (RC), 2.00% pretraining and 1.11% posttraining (WC), 2.50% pretraining and 1.58% posttraining (PC), and 3.29% pretraining and 1.07% posttraining (HC; Figure 1).

### 3.4. Comments from the Observers

After the whole experiment, observers were asked of locations they had difficulty in getting measurements from and its reason why. At least 4 observers answered as follows. (1) Locations of unclear lateral reference points: CW, RW, PW, RC, and PC; (2) location where control of measuring intensity was difficult: IW and CW; (3) location where leveled measuring was difficult: PC.

## 4. Discussion and Conclusions

This study evaluated what extent a comprehensive training improves the reliabilities of the SCBTM which are known to be important factors in the diagnosis of Sasang constitution. 

Pre-training intra- %TEMs of the 5-widths ranged from 1.42% to 6.26%. Interobserver %TEMs ranged from 2.40% to 9.60%. % total TEMs ranged from 4.32% to 10.18% and were in the decreasing order of PW, IW, and CW. Pretraining intra-, inter-observer, and total %TEMs deviation of 5-widths was too large to trust the measurement.

Pre-training intra- %TEMs of the 8-circumferences were in the range of 0.27% to 3.43%. Inter- %TEMs ranged from 1.0% to 2.43%, and % total TEMs were in the range of 1.36% to 3.24%. % total TEMs of 8-circumferences were relatively smaller not only than those in a similar study [22] but also than those of 5-widths in this study, but still somewhat large. As pointed out by % total TEM of widths surging to 10.18%, pretraining results were generally unsuitable. 

After a comprehensive training including a couple of practices intra- and inter-observer reliabilities, hence the % total TEMs were all improved and measurement variation between observers and instructor became smaller. It means that observers' measuring skill has matched the expert's to some degree. 

% total TEMs in both widths and circumferences were all improved to be below 2% except CW (2.18%), which means CW is still hard to measure even after a comprehensive training. CW was also pointed out by the observers as a difficult location to measure. 

There have been fewer studies in width measurement than in circumference. In circumference studies, WHR is widely used. We also checked the WHR using WC and HC and found that a comprehensive training also improved reliability of WHR as shown in previous results [23, 24]. 

Measurement variation was somewhat bigger in the 5 widths than in the 8 circumferences both before and after training. It shows that measurement of width is less consistent than that of circumference. But, 5-width measurements can also be reliable if a more comprehensive practical training is provided. 

In this study, we confirmed that a comprehensive training including practice improved observer's measuring ability. Therefore, a comprehensive practical training should be carried out before collecting the SCBTM data by measurer.

Comments from the observers reveal that it is more difficult to measure width parts than to measure circumference parts. Especially, CW is more difficult to measure for two reasons; one difficulty is to determine lateral standard point and the other is to control the intensity of the measuring instrument. This explains relatively large variation of % total TEM in CW.

Concerning the posttraining 5-width measurements, % total TEMs were within 2.18% and the most reliable variable was IW, followed by RW, WW, PW, and CW. With respect to the 8-circumference measurements, % total TEMs were within 1.58%, FC was the most reliable, followed by CC, HC, WC, AC, RC, NC, and PC. The 8-circumferences measurements are recommended over the 5-width ones for the constitutional diagnosis because of smaller measurement variation. However, it should be considered that how much the measuring location represents the constitutional characteristics. Therefore, further research is necessary to analyze at what degree the 5-widths and the 8-circumferences locations contribute to diagnose Sasang constitution correctly. 

Subjects and observers in this study were only males in their 20s and the sample size is relatively small. How far the repeated measurement training could improve the reliability was not evaluated and follow-up of the training was not carried out to confirm how long the training efforts can last. In spite of these limitations, this study is meaningful in the view point that a comprehensive training makes it more reliable, hence giving a sufficiently confident diagnostic tool to measure the SCBTM and that the observers' measurement skills are close to that of the expert. 

This is the first trial to reveal the reliability of the SCBTM, which is an important factor to diagnose the constitution in the Korean SCM. We also reveal which measurement in width and circumference is more reliable. This study guides the reliability range of the SCBTM of pre- and post-training. It also presents measuring locations in the order of reliability. Authors hope that this study provides foundation for objectification and scientification of the SCM.

##  Conflict of Interests 

The authors declare that there are no competing financial interests.

## Figures and Tables

**Table 1:** A comprehensive training for sasang constitutional body trunk measurement.

Training content	Description
5-width measurement and training	

Observer posture	An observer measures a subject with locating his face in subject's midline.
Subject respiration	A subject maintains stable respiration and measurement is conducted between inspiration and expiration.
Maintenance of horizontal orientation of measuring instruments	Measuring instrument should be located in the same point of the lateral body.
Measuring intensity of measuring instrument	Applied pressure should not distort skin or other human tissue during the measurement.
Measuring method	
Interaxillary width	A subject with attention posture everts left and right arms from the trunk for 5 degrees. An observer places fixed end of the large caliper on the right starting point of subject's axilla. He places movable end of the caliper on the left starting point of subject's axilla and measures the distance between two starting points.
Chest width	A subject with attention posture everts left and right arms from the trunk for 5 degrees. An maginary line is drawn from the right starting point of subject's axilla to lateral corner of the right anterior superior iliac spine (ASIS). This line is superimposed over the line connecting right and left nipples. Fixed end of the caliper is placed on the intersection point of the two lines. Movable end of the caliper is placed on the point contralateral to the intersection point in the left side. Horizontal distance between these two points is measured.
Rib width	A subject with attention posture everts left and right arms from the trunk for 5 degrees. An imaginary line connecting subject's right axillary starting point and distal corner of the right ASIS. Another line connecting right and left 7th and 8th prominence of costochondral junction is drawn. Fixed end of the caliper is placed on the intersection point of the two lines. Movable end of the caliper is placed on the point contralateral to the intersection point in the left side using observer's right hand. Horizontal distance between these two points is measured.
Waist width	A subject with attention posture everts left and right arms from the trunk for 5 degrees. An imaginary line connecting subject's right axillary starting point and distal corner of the right ASIS. Another line connecting right and left Tianshu (ST25) is drawn. Fixed end of the caliper is placed on the intersection point of the two lines. Movable end of the caliper is placed on the point contralateral to the intersection point in the left side using observer's right hand. Horizontal distance between these two points is measured.
Pelvic width	Fixed end of the caliper is placed on the lateral part of subject's right ASIS and movable end of the caliper is placed on the distal of subject's left ASIS. Horizontal distance between these two points is measured.

8-circumference measurement and training	

Observer posture	An observer measures in front of a subject.
Subject respiration	A subject maintains stable respiration and measurement is conducted between inspiration and expiration.
Maintenance of horizontal orientation of measuring instruments	A measuring instrument is placed parallel with observer's hand, eye, and location of the measurement (exception: the shortest distance is measured for the neck circumference).
Measuring intensity of measuring instrument	Applied pressure should not distort skin or other human tissue during the measurement (exception: forehead circumference is measured with finger pressure firmly on subject's hair).
Measuring method	
Forehead circumference	A subject sits up on the chair with his back straightened. An observer holds the “0 point” with one hand. He wraps the tapeline around subject's forehead passing the glabella and the opisthion and overlaps the tapeline and measures. The observer should apply sufficient pressure on subject's hair.
Neck circumference	A subject sits on the chair with his back straightened. He maintains his forehead position parallel to the floor. An observer stands in front of the subject and wraps the measuring tape around the neck passing the area between the thyroid cartilage and the cricoid cartilage. The shortest distance is recorded.
Axillary circumference	A subject undresses upper body and stands up straight with balanced pressure on the right and left feet. An observer stands in front of the subject. The subject raises his arms laterally and the observer wraps measuring tape around the subject's upper body to pass the right and left axilla and midpoint of the chuhndohl (CV22) and the joongjuhng (CV16). The subject lowers their arms in a natural way and the circumference is recorded.
Chest circumference	A subject undresses upper body and stands up straight with balanced pressure on the right and left feet. An observer stands in front of the subject. The subject raises his arms laterally and the observer wraps measuring tape around the subject's upper body to pass the right and left nipple point. The subject lowers their arms in a natural way and the circumference is recorded.
Rib circumference	A subject undresses upper body and stands up straight with balanced pressure on the right and left feet. An observer stands in front of the subject. The subject raises his arms laterally and the observer wraps measuring tape around the subject's upper body to pass the right and left 7th and 8th prominence of costochondral junction. The subject lowers their arms in a natural way and the circumference is recorded.
Waist circumference	A subject undresses upper body and stands up straight with balanced pressure on the right and left feet. An observer stands in front of the subject. The subject raises his arms laterally and the observer wraps measuring tape around the subject's upper body to pass the umbilical cord. The subject lowers their arms in a natural way and the circumference is recorded.
Pelvic circumference	A subject undresses upper body and sufficiently rolls down the pants and underwear to expose measuring areas. He stands up straight with balanced pressure on the right and left feet. The subject crosses his arms on the chest. An observer stands in front of the subject wraps the measuring tape to pass the right and left ASIS. The circumference is recorded.
Hip circumference	A subject undresses upper body and sufficiently rolls down the pants and underwear to expose measuring areas. He stands up straight with balanced pressure on the right and left feet. The subject crosses his arms on the chest. An observer stands in front of the subject wraps the measuring tape to pass right over the pubis. The circumference is recorded.

**Table 2:** General characteristics of the subjects and the observers.

	Subjects	Observers
*N*	10	5
Age (year)	24.4 ± 1.9	25.1 ± 1.5
Weight (kg)	74.16 ± 7.73	67.42 ± 6.89
Height (cm)	178.42 ± 5.96	171.38 ± 1.51

**Table 3:** Pre- and Post-training intraobserver TEM, %TEM, and AB of the measurement.

Location	Observer 1			Observer 2			Observer 3			Observer 4			Observer 5			Instructor	
	TEM	%TEM	AB	TEM	%TEM	AB	TEM	%TEM	AB	TEM	%TEM	AB	TEM	%TEM	AB	TEM	%TEM
IW																	
Pre	0.81	2.54	2.67	0.69	1.97	−0.55	1.32	3.87	0.4	0.96	2.82	0.72	1.61	4.98	2.17	0.25	0.72
Post	0.63	1.85	0.36	0.27	0.79	0.07	0.39	1.15	0.22	0.51	1.49	0.23	0.3	0.88	0.42	0.36	1.05
CW																	
Pre	0.63	2.11	2.47	2.03	6.26	0.02	0.82	2.51	−0.13	0.59	1.91	1.62	0.83	2.74	1.99	0.39	1.2
Post	0.48	1.52	0.6	0.46	1.42	−0.37	0.33	1.05	0.46	0.5	1.6	0.71	0.43	1.36	0.68	0.26	0.81
RW																	
Pre	0.61	2.18	1.99	0.98	3.39	1.2	1.02	3.48	0.77	0.42	1.42	0.92	0.67	2.32	1.25	0.34	1.12
Post	0.23	0.77	0.36	0.36	1.22	0.1	0.24	0.8	−0.12	0.41	1.38	0.23	0.43	1.47	0.44	0.24	0.8
WW																	
Pre	0.74	2.78	2.81	0.47	1.64	0.59	0.55	1.9	0.43	0.54	1.92	1.22	0.59	2.11	1.38	0.17	0.57
Post	0.41	1.42	0.35	0.26	0.87	0.03	0.29	0.98	−0.05	0.22	0.77	0.32	0.5	1.72	0.53	0.21	0.72
PW																	
Pre	1.02	3.62	−1.03	0.91	2.85	−4.67	1.41	5.71	2.5	0.66	2.37	−0.78	0.59	1.99	−2.31	0.22	0.81
Post	0.32	1.2	0.32	0.38	1.37	−0.41	0.24	0.9	−0.08	0.34	1.26	−0.1	0.16	0.58	0.4	0.2	0.74

FC																	
Pre	0.16	0.27	−1.54	0.57	0.97	−1.39	0.52	0.9	−0.7	0.79	1.35	−1.12	0.49	0.82	−1.85	0.23	0.4
Post	0.25	0.44	−0.08	0.17	0.3	−0.63	0.31	0.54	−0.7	0.17	0.29	−0.54	0.15	0.27	−0.44	0.24	0.42
NC																	
Pre	0.39	1.04	0.18	1.03	2.69	−0.66	0.81	2.1	−0.95	0.89	2.38	0.21	0.6	1.54	−1.28	0.3	0.8
Post	0.35	0.93	0.02	0.28	0.74	0.1	0.37	0.98	−0.55	0.3	0.79	0.17	0.32	0.86	−0.28	0.28	0.76
AC																	
Pre	1.34	1.42	2.99	2.02	2.1	1.67	1.47	1.49	−0.86	1.67	1.73	1.18	1.06	1.09	−0.22	0.48	0.49
Post	0.65	0.66	−0.65	0.45	0.46	1.03	1.02	1.03	−0.8	0.5	0.51	0.43	0.57	0.58	0.35	0.57	0.58
CC																	
Pre	1.04	1.16	3.6	1.31	1.44	2.55	1.19	1.28	0.98	2.01	2.2	2.2	1.17	1.28	1.68	0.55	0.59
Post	0.5	0.54	0.82	0.63	0.67	0.7	0.51	0.55	−0.01	0.38	0.41	0.58	0.47	0.5	0.95	0.46	0.49
RC																	
Pre	3.04	3.81	2.31	2.37	2.96	1.98	1.03	1.28	1.81	1.5	1.86	1.21	1.12	1.36	0.35	0.45	0.55
Post	0.83	1.01	0.27	0.57	0.7	0.6	0.45	0.54	−0.52	1.11	1.35	−0.42	0.75	0.91	0.44	0.48	0.59
WC																	
Pre	1.3	1.62	1.91	0.94	1.14	0.03	1.29	1.56	−0.28	0.74	0.91	0.58	1.17	1.42	−0.26	0.35	0.42
Post	0.68	0.83	0.15	0.47	0.57	0.23	0.65	0.78	−0.84	0.48	0.59	0	0.53	0.64	−0.19	0.56	0.68
PC																	
Pre	0.94	1.12	2.58	0.73	0.87	1.55	1.04	1.24	1.46	1.74	2	−0.99	1.15	1.35	0.86	0.74	0.86
Post	0.41	0.48	1.23	0.61	0.71	0.79	0.95	1.1	0.15	0.82	0.94	−0.1	0.76	0.87	−0.8	0.83	0.96
HC																	
Pre	1.5	1.68	2.63	1.65	1.8	−0.12	1.2	1.27	−2.37	3.19	3.43	−1.16	2.06	2.24	−0.11	0.53	0.58
Post	0.53	0.57	0.05	0.35	0.38	0.05	0.93	0.99	−1.4	0.44	0.47	−0.32	0.33	0.35	−0.58	0.49	0.53
WHR																	
Pre	0.02	2.12	−0.01	0.02	2.4	0.001	0.02	2.18	0.02	0.03	3.1	0.02	0.02	2.39	−0.002	0.01	0.76
Post	0.01	1.05	0.001	0.006	0.7	0.002	0.007	0.77	0.004	0.006	0.7	0.003	0.007	0.81	0.004	0.009	1.05

TEM: technical error of measurement, AB: mean difference between measurement of observer and that of the instructor.

**Table 4:** Pre- and post-training interobserver TEM and % TEM of the measurement.

Location	Pretraining		Posttraining	
	TEM	%TEM	TEM	%TEM
IW	1.519	4.53	0.279	0.82
CW	1.318	4.22	0.528	1.66
RW	0.693	2.40	0.334	1.12
WW	1.067	3.78	0.385	1.31
PW	2.732	9.60	0.441	1.62
FC	0.587	1.00	0.327	0.56
NC	0.832	2.19	0.406	1.08
AC	1.732	1.80	1.032	1.05
CC	1.278	1.40	0.787	0.85
RC	1.413	1.75	0.808	0.98
WC	1.200	1.47	0.714	0.87
PC	1.767	2.08	1.149	1.33
HC	2.244	2.43	0.816	0.88
WHR	0.018	2.08	0.008	0.92

TEM: technical error of measurement.
